# Effect of Metallurgical Process on Rotational Bending Fatigue Properties of H13 Hot Work Die Steel

**DOI:** 10.3390/ma18245655

**Published:** 2025-12-16

**Authors:** Yunling Li, Dangshen Ma, Shulan Zhang, Xiaofei Sun, Yuan Li, Zijian Zhang, Zhenqian Zhong

**Affiliations:** 1 Central Iron & Steel Research Institute Co., Ltd., Beijing 100081, China; liyunling@ncschina.com (Y.L.);; 2 NCS Testing Technology Co., Ltd., Beijing 100081, China; 3 Power E-Procurement (Beijing) Technology Co., Ltd., Beijing 100080, China

**Keywords:** H13, rotational bending fatigue, metallurgical process, fracture characteristic, fracture mechanism

## Abstract

A series of high-cycle rotating-bending fatigue tests was conducted on H13 steel produced by electroslag remelting (ESR) and by vacuum induction melting followed by vacuum arc remelting (VIM+VAR). At 10^7^ cycles, the fatigue strength of VIM+VAR steel was 1040 MPa, which is greater than the 967 MPa of ESR steel. A metallographic analysis was conducted to compare the structure and grain size of the two steels. The results indicated that while the two steels were similar, ESR steel contained a greater number of larger inclusions and carbides. The mean inclusion size in VIM+VAR steel was approximately 55% of that in ESR steel, and the maximum inclusion size was around 44%. Notwithstanding this finding, the fatigue strength of VIM+VAR steel was found to be approximately 7.5% higher. Scanning electron microscopy of fracture surfaces revealed that the primary cause of crack initiation was predominantly oxides or oxide-sulfide composites. The measurements obtained for inclusion size, fisheye diameter, and crack propagation length indicated that the fatigue life of the material is governed primarily by the applied stress and the size of the inclusion. The presence of larger inclusions has been demonstrated to reduce the crack-propagation stage and decrease the steel’s tolerance to defects, thereby reducing fatigue life and endurance limit. The researchers derived formulae relating inclusion size to stress intensity factor and fatigue life by utilizing the Paris law. These equations ·the fatigue-fracture mechanism and provided a basis for predicting the rotating-bending fatigue life of H13 steel.

## 1. Introduction

H13 is a martensitic hot work die steel that exhibits ideal tempering resistance, good thermal fatigue resistance, excellent thermal crack resistance, and high hardness and strength at elevated temperatures. Its utilization is extensive, being employed in precision forging dies for gears [1], hot extrusion dies for pipeline steel [2], aluminum alloy die-casting molds [3], and components of tunnel boring machines [4]. Its utilization within the domain of advanced manufacturing has become imperative, as evidenced by its presence in the automotive, petrochemical, aerospace, and rail transportation industries [5,6,7].

In order to enhance the properties of H13 steel, comprehensive research has been conducted on the optimization of alloy composition [8] and the control of heat treatment. As Wang Wen et al. [9] have previously demonstrated, an augmentation in both the pre-tempering temperature and the holding time has been shown to result in an increase in martensite decomposition, an enlargement of carbide size, a reduction in hardness, and an increase in impact energy. Di Yingnan [10] demonstrated that cryogenic treatment enhances strength and toughness without compromising plasticity in comparison with conventional heat treatment. It is evident that the performance of H13 is subject to variation in relation to the processes of smelting and heat treatment [11]. The presence of inclusions and carbides, in conjunction with the subsequent microstructure that results from post-treatment processes, has been demonstrated to exert a substantial influence on the properties of the material [12]. However, the disparities in inclusion and carbide type, distribution, and quantity between various smelting processes remain unreported.

During the component’s service life, H13 steel is subjected to cyclic stress and thermal shock, resulting in local plastic deformation and fatigue failure [13,14,15]. A range of strategies has been investigated with a view to enhancing fatigue performance and service life. Lin Huangxu et al. [16] developed a Johnson–Cook constitutive model for H13 steel. This was achieved by means of orthogonal cutting tests and finite element simulations. Dai Shangyi et al. [17] established a crystal plasticity finite element model coupling dislocation density and damage evolution to reveal fracture mechanisms during room-temperature tensile testing. Silvestri A. [18] investigated the effects of laser-directed energy deposition parameters on single-layer H13 quality, finding that unidirectional scanning with 60% overlap minimizes waviness and improves surface uniformity by ~25% compared to bidirectional strategies. In the study by Zhou Hu et al. [19], the application of laser cladding was utilized to form Stellite 6 and Y_2_O_3_/Stellite 6 composite coatings on forging die cavities. The resultant coatings exhibited superior thermal fatigue resistance in comparison to the H13 substrate. In the study by Shao Yuhang et al. [20], CrAlN/TiAlN composite coatings were prepared on nitride H13 steel via multi-arc ion plating. The resultant coatings demonstrated a 20 times increase in hardness and a two-order of magnitude reduction in wear rate compared to the substrate. Liao Yongfa et al. [21] formed iron nitride diffusion layers on H13 steel through hollow cathode ion source nitrocarburizing, significantly improving hardness and wear resistance. The present studies on H13 steel performance are chiefly concentrated on tensile properties, thermal fatigue, and wear resistance. However, the rotating bending fatigue behavior—which is critical for structural components—remains unaddressed.

The present study investigates the influence of smelting processes on the fatigue strength and life of H13 steel through rotating bending fatigue tests. Two types of H13 steels were utilized in the study: one produced by electroslag remelting (ESR) and the other by vacuum induction melting followed by vacuum arc remelting (VIM+VAR). The investigation into the influence of smelting processes on inclusion and carbides in H13 steel is conducted through two complementary approaches. Firstly, the qualitative characterization of inclusions at fatigue fracture surfaces is undertaken using scanning electron microscopy (SEM). Secondly, the quantitative characterization of inclusions and carbides in the matrix is conducted utilizing ASPEX. An analysis was conducted to ascertain the effects of inclusion, carbide size, and quantity on rotating bending fatigue life. The Paris crack propagation formula was finally applied in order to theoretically examine the relationship between inclusion size, alternating stress, fatigue life, and fatigue strength. The objective of this study was to propose innovative strategies for predicting the fatigue performance and service life of H13 steel.

## 2. Materials and Methods

### 2.1. Material Preparation

The experimental material utilized was H13 hot work die steel. Steel A was subjected to electroslag remelting (ESR), while Steel B was subjected to a combination of vacuum induction melting and vacuum arc remelting (VIM+VAR).

The production process of Steel A is as follows: electric arc furnace melting, ladle furnace refining, vacuum degassing treatment, casting molding, and ESR. The diameter of the electroslag furnace crystallizer used for ESR is 380 mm, and the CA73 slag, consisting of 70% wt CaF2 and 30% wt Al_2_O_3,_ is used for remelting. The remelting current is 23,000~28,000 A. During the remelting process, a thermocouple is used to measure slag pool temperature, and the remelting time is about 210 min. The electroslag ingot, weighing about 12.5 tons, is obtained after remelting and demolding.

In order to remove O, N, and other impurities in the material, Steel B was melted by the VIM+VAR process, aiming to improve the mechanical properties. The vacuum is better than 10^−4^ Torr. VIM melting temperature is 1450 °C, and VAR refining temperature is 1600 °C. A remelting ingot of 890 mm diameter and a consumable ingot of 1050 mm diameter are used for VAR refining for 2 h. The VIM+VAR ingot weighs about 12.2 tons.

Subsequent metrology revealed that both ingots were indistinguishable in terms of their physical dimensions, exhibiting a diameter of 305 mm. Subsequent to a process of homogenization at a temperature of 1250 °C for a duration of 45 h, the samples were subjected to forging and rolling, resulting in the formation of bars measuring 20 mm in diameter. The chemical compositions are listed in Table 1. A comparison of the two steels reveals a similarity in their composition, with Steel B exhibiting significantly lower concentrations of phosphorus (P), nitrogen (N), and total oxygen (T.O) compared to Steel A.

**Figure 1:**
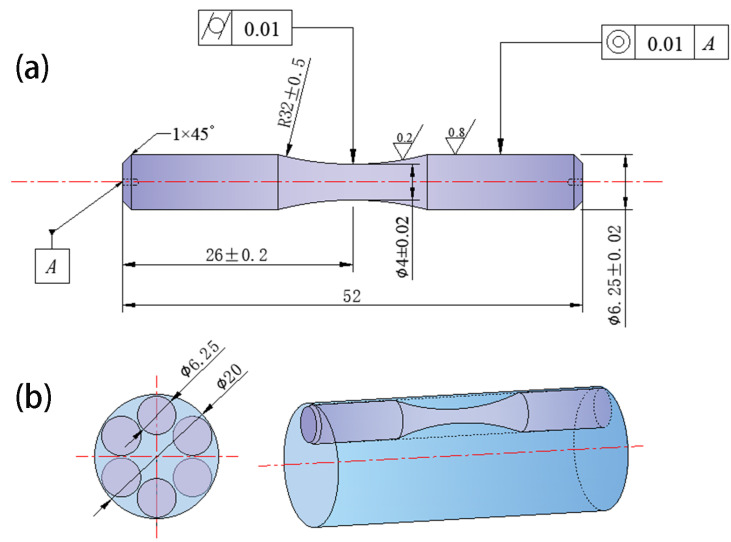
Sample collection diagram: (**a**) sample dimensional drawing, (**b**) sampling position.

Samples were wire-cut from Steel A and B, respectively: a 10 mm × 10 mm × 0.3 mm foil for transmission electron microscope (TEM), a 10 mm × 10 mm × 20 mm longitudinal metallographic block, a 20 mm × 20 mm × 10 mm phase-analysis specimen, an M12 × 65 mm tensile bar, and 30 blanks for high-cycle rotating-bending fatigue tests. The fatigue blanks were then subjected to turning, heat treatment, and finish-grinding to standard dimensions; the sampling layout is shown in Figure 1. The two steels were subjected to an identical heat treatment: The initial step is to preheat the material to 750 °C, maintaining this temperature for a period of 45 min. Following this, the material should be held at 1000 °C for 30 min in order to undergo a process known as austenitization. Subsequently, the material should be oil-cooled to room temperature prior to undergoing deep-cooling at −80 °C for a duration of 1 h. This is then followed by heating the material to 400 °C, where it should be left for a period of 2 h in order to undergo the process of tempering. Finally, the material should be air-cooled, and it should be noted that a total of two tempering cycles is required.

### 2.2. Microstructure, Inclusion, and Phase Analyses Test

Longitudinal metallographic specimens were subjected to grinding from 120# to 1500# SiC paper, and subsequently polished with 2.5 µm diamond paste. The inclusions were then subjected to automatic analysis on a Thermal Particle X SEM (Thermo Fisher Scientific, Waltham, MA, USA), utilizing predefined parameters. The accelerating voltage is 15 kV, the working distance is 8.5 mm, the spot is 60 mA, and the scan area is 160 mm^2^. Subsequent to the etching process with 4% nitric acid, the matrix was subjected to a detailed examination using Olympus optical microscopy (Olympus Corporation, Hachioji-shi, Tokyo, Japan). The characterization of grain size and low- to high-angle boundaries was conducted on a JEOL SEM equipped with an Oxford Symmetry electron backscatter diffraction (EBSD) detector (Oxford Instruments, Abingdon, TW, UK).

The TEM specimen was manually ground with a grinding disk to a thickness of less than 100 μm, and then the thin sheets were punched into multiple disks measuring 3 mm in diameter and of the original thickness. The disk TEM sample was thinned by twin-jet electropolishing [22] in 10% perchloric acid at 20 kV using a Fischione 110 unit (Fischione Instruments, Export, PA, USA) until perforation. The foils were examined using a Talos F200X G2 TEM (Thermo Fisher Scientific, Waltham, MA, USA) to observe the morphology and distribution of the carbides. The carbide chemistry was determined by means of energy-dispersive X-ray spectroscopy (EDS), and the carbide type was identified by combining EDS data with selected-area electron diffraction (SAED) patterns.

The electrolysis extraction method has been demonstrated to be a valuable technique for the electrolytic extraction of fine carbides from steel specimens for subsequent XRD testing. This approach enables both quantitative and qualitative analysis of carbides in steel, facilitating a comprehensive assessment of the material’s composition [23]. Electrolytic extraction experiments were conducted on experimental steel materials with different smelting processes at an electrolysis temperature ranging from −5 °C to 0 °C. The electrolyte composition comprised citric acid, methanol, hydrochloric acid, and glycerol. Carbide powders were obtained and analyzed for phase structure using an X-ray diffractometer Xeuss 3.0 C (Xenocs, Nanometer, Grenoble, France) under the following conditions. The target parameters for this experiment are as follows: a working current and voltage of 40 mA and 35 kV, respectively, a scanning range of 20° to 115°, and a scanning rate of 0.02°/s.

### 2.3. Mechanical Property Test

Tensile tests were conducted at ambient temperature on a universal testing machine, MTS Criterion40 (MTS system, Eden Prairie, MN, USA), at a rate of 0.5 mm/min, in accordance with standard GB/T 228.1-2021 [24]. Rotating bending fatigue tests were conducted on a QBWP-10000 machine (Changchun Qianbang, Changchun, Jilin, China) in accordance with standard GB/T 4337-2015 [25]. The operating frequency of the machine was set at 80 Hz, and stress ratio R = −1 was utilized. S-N curves were plotted from recorded stress and life data. The fracture surfaces were examined by means of SEM. The following parameters were measured: inclusion size (*d_inc_*), inclusion depth (*h_inc_*), fisheye diameter (*2b*_1_), and propagation zone length (*d*_3_). A comparative analysis was conducted on the fracture parameters and stress intensity factors of the two steels in order to ascertain the impact of inclusion/carbide type, location, and size on their rotating bending performance.

## 3. Results

### 3.1. Microstructure and Grain Size

Grain size is a pivotal factor in determining steel strength, and grain refinement is a prevalent technique employed to enhance both strength and toughness [26]. In addition to mechanical properties, the effect of grain size on rotating bending fatigue performance is also significant. To illustrate this point, consider the example of refined bearing steel, which exhibits a median fatigue strength that is 30 MPa higher than that of the original material [27].

**Figure 2:**
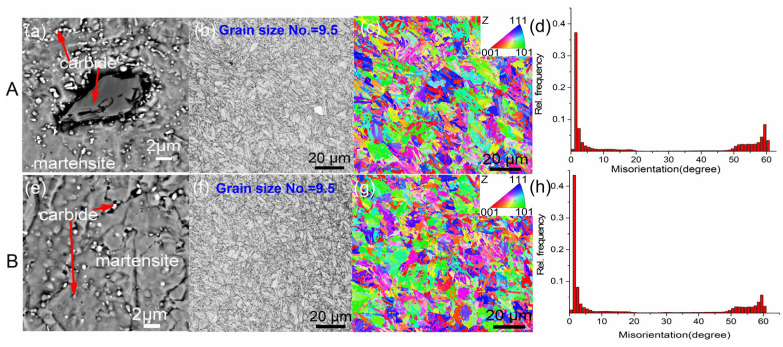
Metallographic structure and grain size of steel A, B: (**a**,**e**) metallographic structure, (**b**,**f**) grain size, (**c**,**g**) EBSD IPF map, (**d**,**h**) EBSD misorientation distribution map.

As illustrated in Figure 2, the metallographic structure and grain size of steels A and B are presented. The SEM images reveal the presence of carbides in steel A, which manifest as both black blocky and white particulate types (Figure 2a). In contrast, only white particulate carbides are observed in steel B (Figure 2e). Optical microscopy reveals that the grain sizes of both steels are 9.5 (Figure 2b,f). As demonstrated in the IPF maps (Figure 2c,g), there is an absence of preferred textures, with uniform orientations being exhibited [28]. Furthermore, the misorientation distributions (Figure 2d,h) demonstrate comparable characteristics. The results of this study indicate the existence of microstructural differences, which are primarily attributable to carbide characteristics. In order to elucidate the disparities in carbide type, distribution, and content between the two steels, the following methodology is recommended. The use of TEM is recommended for the observation of the morphology of carbides, while SAED and EDS should be employed for the determination of the type of carbides. Concurrently, the utilization of electrolytic extraction and XRD is imperative for the quantitative analysis of the content and composition of carbides within the alloy.

### 3.2. Carbide

**Figure 3:**
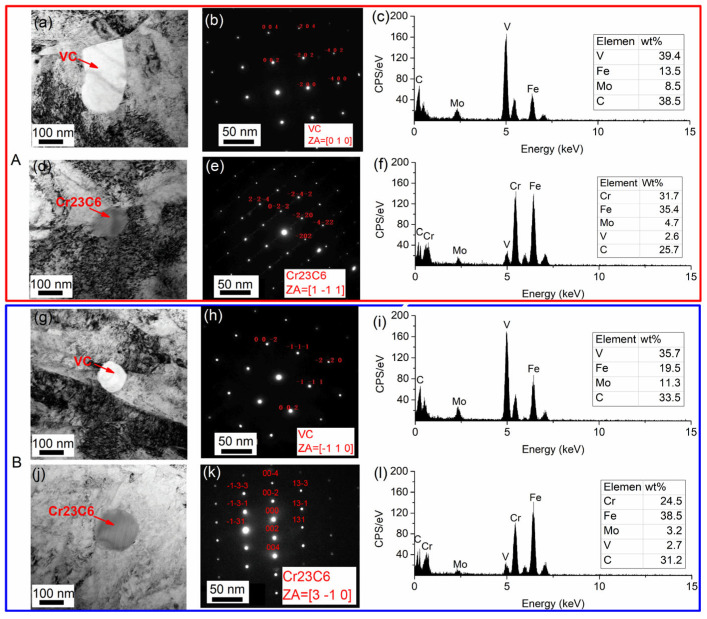
Carbides in steel A, B: (**a**,**d**,**g**,**j**) TEM morphology; (**b**,**e**,**h**,**k**) SEAD pattern; (**c**,**f**,**i**,**l**) EDS spectrum.

As demonstrated in previous studies [29,30,31], there is a significant variation in carbide types present within H13 steel, which is contingent upon the processing conditions employed. As demonstrated in Figure 3, the TEM images, SAED patterns, and EDS results of the precipitates are presented. Bright white square and spherical particles, marked by arrows in Figure 3a,g, measure 140~320 nm. EDS analysis has been used to reveal the presence of V, Fe, Mo, and C in these particles. As illustrated in Figure 3b,h, the SAED patterns correspond to VC (FCC, a = 0.416 nm) along [010] and [−110] zone axes. Consequently, the precipitates have been identified as V-rich MC carbides, which are characterized by a face-centered cubic structure.

The second type is constituted of dark gray spherical precipitates, with a size of approximately 150~200 nm, as illustrated in Figure 3d,j. EDS analysis (Figure 3f,l) indicates elevated concentrations of Cr and Fe, along with Mo, V, and C. SAED patterns (Figure 3e,k) correspond to those of Cr23C6 (FCC, a = 1.069 nm) across the [1–11] and [3–10] zone axes. These precipitates are identified as Cr-rich M23C6 carbides with a face-centered cubic structure.

**Figure 4:**
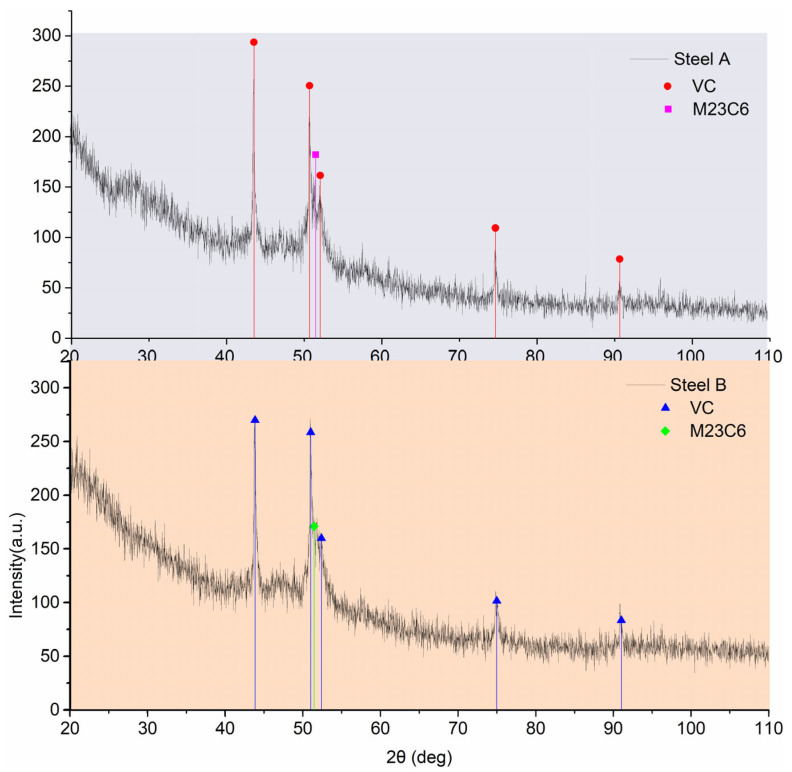
Phase analysis X-ray diffraction spectrum.

As illustrated in Figure 4, the XRD patterns of electrolytically extracted precipitates from steels A and B reveal the presence of the primary phases VC and M23C6, which is in alignment with the results obtained through TEM analysis. Quantitative analysis (see Table 2) indicates a decrease in the mass fraction of MC carbides from 1.176% in steel A to 1.141% in steel B, while M23C6 carbides increase from 1.010% to 1.015%.

It is evident from the analysis of grain size, microstructure, TEM, and phase analysis that the two steels exhibit similar grain size and microstructure, with identical carbide types (MC and M23C6). However, a divergence in their carbide contents is observed: It is evident that Steel A contains a greater quantity of MC, yet it possesses a lower amount of M23C6 in comparison to Steel B. The limitations imposed by the available observation areas, in conjunction with the phase analysis’s incapacity to ascertain carbide morphology, have resulted in the inability to obtain quantitative characterization of carbide size and distribution. Accordingly, the Thermal Particle X SEM (Thermo Fisher Scientific, Waltham, MA, USA) was utilized to analyze the mean size and distribution of inclusions and carbides in the two steels.

### 3.3. Characterization of Inclusion and Carbide Size

**Figure 5:**
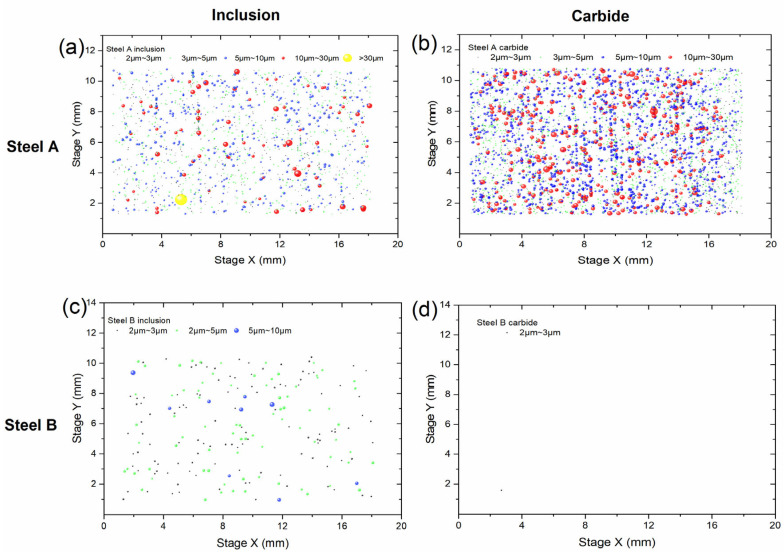
Inclusion and carbide distribution of steel A and B: (**a**,**c**) inclusion distribution of Steel A or B; (**b**,**d**) carbide distribution of Steel A or B.

Inclusions have been demonstrated to exert a substantial influence on the rotating bending fatigue performance of materials [32], and the control of carbide precipitation has been demonstrated to optimize the microstructure of H13 steel, thereby achieving excellent mechanical properties [33]. It is therefore essential to compare the inclusion and carbide levels in steels A and B, which were produced by different smelting processes but underwent the same heat treatment. The characterization results of inclusions and carbides in the two steels are shown in Figure 5. Steel A contains a high number of inclusions and carbides larger than 10 µm, while Steel B has very few carbides and only a small number of inclusions between 2 and 10 µm. As illustrated in Table 3, the statistical results of average size and number density of inclusions and carbides are presented. The area number density of inclusions and carbides larger than 2 µm in Steel A is significantly higher than in Steel B, with inclusions being approximately four times and carbides more than twenty times higher. In addition to their dense distribution, the average Feret diameter of inclusions and carbides in Steel A is similar, at approximately 4.5 µm, which is larger than the approximately 3 µm in Steel B.

### 3.4. Mechanical Properties and Rotary Bending Fatigue Performance

**Figure 6:**
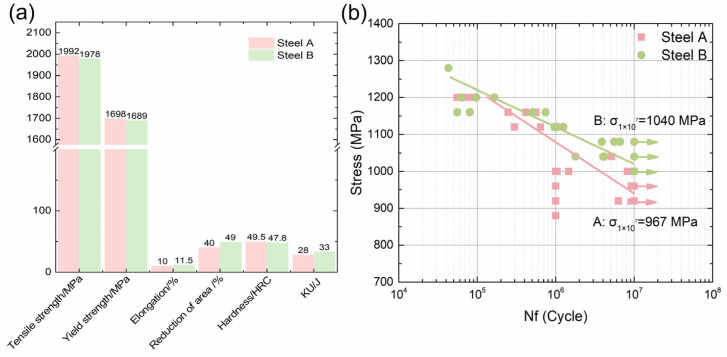
Mechanical properties of steel A and B: (**a**) tensile, hardness and Charpy test result; (**b**) rotary bending fatigue test result; Steel A Fit line: log(*Nf*) = 57.41 − 16.96 × log(Stress), Pearson’s R: −0.9153); Steel B Fit line: log(*Nf*) = 85.39 − 26.06 × log(Stress), Pearson’s R: −0.7171).

The fundamental mechanical properties of the two steels are illustrated in Figure 6. As demonstrated in Figure 6a, Steel A demonstrates higher tensile strength, yield strength, and hardness in comparison to Steel B. In contrast, Steel B exhibited enhanced elongation, reduced area, and increased impact energy. Its room-temperature U-notch impact energy (KU) was measured at 33 J, which is approximately 18% higher than the 28 J recorded for Steel A. As illustrated in Figure 6b, the rotating-bending S-N curves obtained by the multi-specimen method are presented. As the applied stress is reduced, the cycle count increases, and a fatigue limit plateau becomes apparent. Utilizing the staircase method, the rotating-bending fatigue strengths at 10^7^ cycles (σ_1×10_^7^) are determined to be 967 MPa for Steel A and 1040 MPa for Steel B. Consequently, the fatigue strength of Steel B is approximately 7.5% higher than that of Steel A.

### 3.5. Characteristics of Rotary Bending Fatigue Fracture

**Figure 7:**
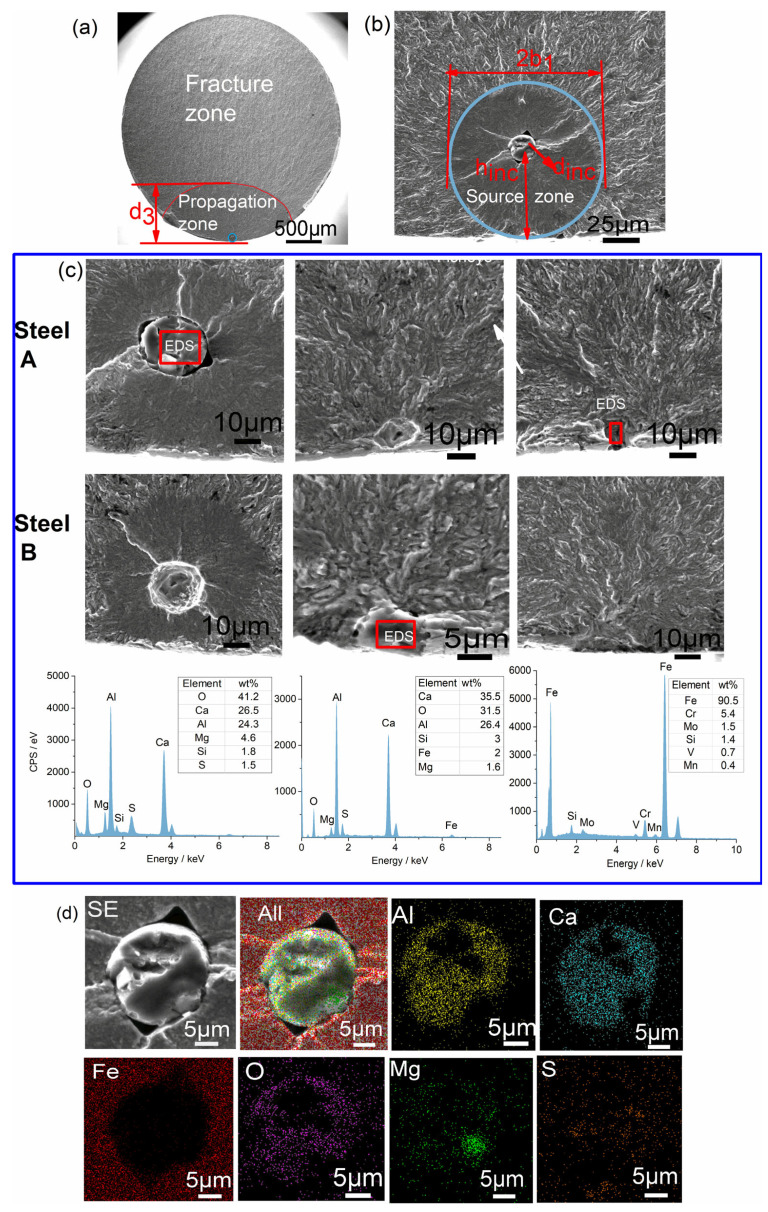
Morphology of fracture and EDS analysis of source zone: (**a**) fracture morphology; (**b**) source zone morphology; (**c**) EDS spectrum of source zone; (**d**) EDS map of inclusion.

Following ultrasonic cleaning, the fracture surfaces of the rotating-bending fatigue specimens were examined by SEM. As illustrated in Figure 7a,b, the fracture morphology can be categorized into three distinct zones: source, propagation, and fracture. The following parameters were measured: *d_inc_* (square root of the projected inclusion area at the fracture plane), *h_inc_* (distance from the inclusion center to the specimen surface), *2b*_1_ (tangential length of the fisheye), and *d*_3_ (radial length from the surface to the front of the final-fracture zone).

As demonstrated in Figure 7c, the crack origins of steel A and B can be classified into two distinct categories. The initial type is located at the periphery of the sample, exhibiting a composition that is consistent with the matrix and demonstrating an absence of defects in the source region. The second type contains complex inclusions of Al, Ca, Mg, S, O, and other elements, located at the edge or within the sample. As illustrated in Figure 7d, the use of EDS mapping provides confirmation of the complex composition. As reported in previous studies [34], characteristic zones are observed around matrix or inclusion origins. These zones are optically dark areas (ODA) under light microscopy and granular bright facets (GBF) under SEM. It has been determined that fissures propagate in these areas, forming what is colloquially referred to as ‘fisheyes’. Research has demonstrated that fisheye growth is responsible for 80–90% of the total fatigue life [35], and it has been established that higher purity and smaller inclusions result in greater fatigue strength [36,37]. The smooth origin region depicted in Figure 7b is attributed to repeated tension-compression cycles, whereby crack advancement and closure alternate [38,39]. The mechanisms by which these phases form remain the subject of debate, including the role of hydrogen-assisted initiation [40,41], the involvement of carbide [38], and the competition between local stress intensity [42]. The fracture parameters employed in this study include the inclusion size (*d_inc_*), depth (*h_inc_*), fisheye diameter (*2b*_1_), and final-fracture front depth (*d*_3_). The utilization of these four fracture parameters facilitates the correlation of fracture features with failure characteristics.

**Figure 8:**
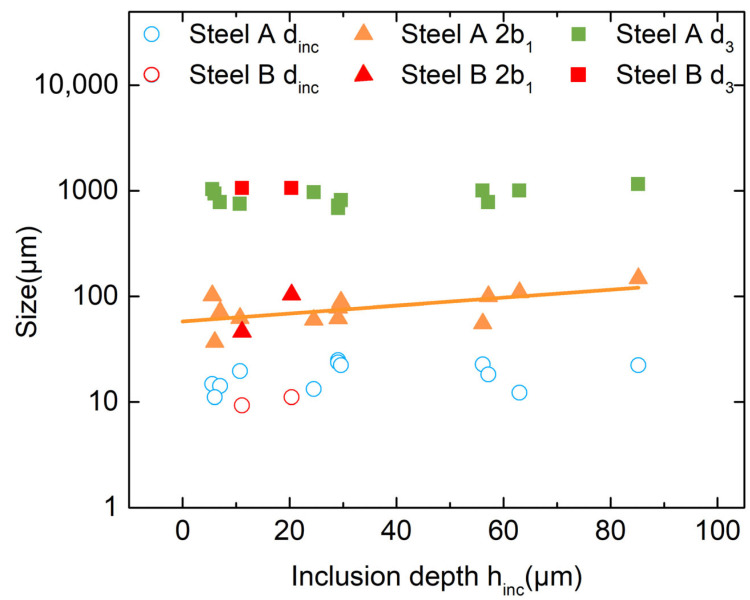
Relationship between fracture characteristic parameters (Fit line: *2b*_1_ = 1.76 + 0.0036 × *h_inc_*, Pearson’s R: 0.6172).

Inclusion size is widely regarded as a key factor affecting fatigue life, with the maximum or average inclusion size significantly influencing fatigue performance [43]. As demonstrated in Figure 8, the ranges of *d_inc_* and *h_inc_* in Steel A are 11~25 μm and 6~85 μm, with average values of 18 μm and 33 μm, respectively. In contrast, Steel B displays a range of 9~11 μm for *d_inc_* and 5~20 μm for *h_inc_*, with averages of 10 μm and 16 μm, respectively. This finding indicates that the mean inclusion size in Steel B is approximately 55% of that in Steel A, with a maximum inclusion size of around 44%. Notwithstanding this observation, Steel B demonstrates a fatigue strength of 1040 MPa, which is approximately 7.5% higher than that exhibited by Steel A. These results also suggest that inclusions or carbides larger than 10 μm significantly affect fatigue life. As demonstrated in Figure 5, Steel A exhibits a significantly higher number of inclusions and carbides exceeding 10 μm in comparison to Steel B. This observation indicates that the enhanced fatigue strength of Steel B is associated with its reduced content of inclusions and carbides larger than 5 μm.

As demonstrated in Figure 8, no discernible correlation is evident between *d_inc_* or *d*_3_ and *h_inc_*. However, 2*b*_1_ demonstrates a robust linear relationship with *h_inc_*—fisheyes undergo expansion as inclusion depth rises. It can be concluded that, given the established correlation between the fisheye size and crack growth capacity, the inclusion distribution exerts a substantial influence on crack propagation during the process of rotating bending fatigue.

The findings indicate that factors such as inclusion type, distribution, and size have a significant impact on fatigue performance. In conjunction with the characterization of inclusions outlined in Section 3.3, the fracture-initiating inclusions in both steels are predominantly oxide-sulfide composites. It is evident that the size of the fisheye increases with the depth of inclusions. However, it is worth noting that there are more and larger inclusions at the origin of the crack in Steel A. This results in a lower fatigue strength in comparison to Steel B.

## 4. Discussion

### 4.1. Relationship Between Fracture Characteristic Parameters, Fatigue Life, and Applied Stress

**Figure 9:**
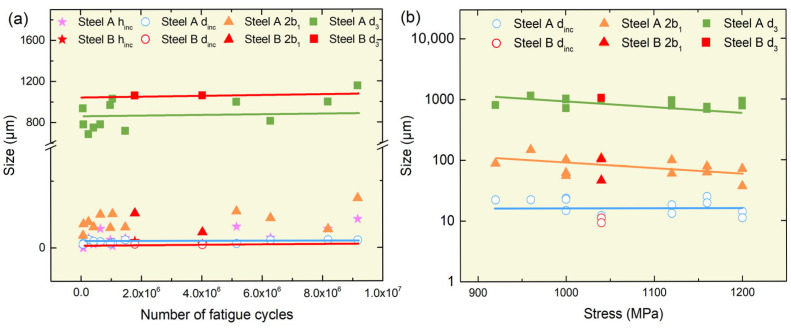
Relationship between fracture parameters, fatigue life, and applied stress: (**a**) size and fatigue life; (**b**) size and stress. (**a**) Fit line Steel A: *d_inc_* = 5.31 × *N_f_* + 16.76, Pearson’s R: 0.3657; *d*_3_ = 2.65 × *N_f_* + 811, Pearson’s R: 0.6058; Fit line Steel B: *d_inc_* = −8.28 × *N_f_* + 12.59, Pearson’s R: −1; *d*_3_ = −4.47 × *N_f_* + 1070, Pearson’s R: −1; (**b**) Fit line *d*_3_ = −3 × Stress + 3.26, Pearson’s R: −0.4036; *2b*_1_ = −8.53 × Stress + 2.80, Pearson’s R: −0.5166; *d_inc_* = −5.61 × Stress + 1.85, Pearson’s R: −0.4416.

As illustrated in Figure 9a, a correlation exists between the fatigue fracture parameters and the fatigue life of steel A and B. The *d_inc_* and *d*_3_ demonstrate negligible variation with fatigue life, thus serving as the minimum and maximum crack sizes, respectively. For Steel A, the *d_inc_* is approximately 18 μm, and the *d*_3_ is approximately 885 μm; for Steel B, the *d_inc_* is about 10 μm, and the *d*_3_ is approximately 1063 μm. This finding suggests that steel B exhibits a wider minimum–maximum crack range, indicating a higher tolerance to crack propagation length.

Figure 9b shows that a clear correlation exists between the fracture parameters and the applied stress for both steels. As the magnitude of the applied stress increases, two phenomena become observable: firstly, a decrease in the *2b*_1_; secondly, a decrease in the *d*_3_. Concurrently, *d_inc_* remains unchanged with stress. It has been established that, during the testing process, the largest inclusions located in close proximity to the surface undergo continuous cycles of tension and compression. This results in a gradual expansion of the fisheye zone. This zone appears to be relatively smooth [34,44]. Upon contact between the fisheye and the specimen surface, instantaneous fracture occurs. At the moment of tearing, the external load continues to act on the specimen and influences the size of the final fracture zone [45].

The analysis demonstrates that inclusion size and the depth of the final-fracture front can be regarded as the minimum and maximum crack sizes during fatigue fracture, respectively. These parameters remain constant with the cycle count. The present study has failed to establish a definitive correlation between fracture feature dimensions and fatigue life. Furthermore, the relationship between feature dimensions and applied stress is characterized by significant scatter. In order to achieve a more profound comprehension of these relationships, it is necessary to consider the intrinsic interactions among stress, fatigue life, and characteristic parameters. Moreover, the maximum tolerable crack size is greater in Steel B than in Steel A. When the results of Figure 5 are taken into consideration, the maximum inclusion size in Steel B is smaller than in Steel A. This indicates that larger inclusions reduce the steel’s tolerance to crack size, thereby lowering its fatigue life and endurance limit.

### 4.2. Fracture Mechanics of Crack Propagation

It has been established that significant disparities exist between the elastic modulus and hardness of inclusions and the metal matrix [46]. Consequently, fatigue cracks gradually initiate at the interface between the inclusions and the matrix under repeated axial tension–compression loading. The stress state in the vicinity of the crack tip is determined by the stress intensity factor [47]. In accordance with the principles of fracture mechanics theory [48], the relationship between the stress intensity factor and fracture characteristic parameters is expressed by the following Equation (1):

(1) $$\Delta K=Y\sigma \sqrt{\pi D}$$

The shape factor Y is specified as 0.65 for surface inclusions and 0.5 for internal inclusions [49].

Given the tangency of the fisheye region to the fracture surface, the applied stress can be considered to act directly on the fisheye and propagation zone. Substituting parameters into Equation (1) yields Equations (2) and (3):

(2) $$\Delta k_{fisheye}=0.5\sigma \sqrt{\pi {2b}_{1}}$$

(3) $$\Delta k_{propagation}=0.5\sigma \sqrt{\pi d_{3}}$$

In these equations, *2b*_1_ denotes the diameter of the fisheye, and *d*_3_ is defined as the radial distance measured from the specimen surface to the front of the final fracture zone. This phenomenon is analogous to that depicted in Figure 7a,b.

Furthermore, the majority of inclusions observed within the fracture surfaces are of an internal and spherical nature. Substitution of the inclusion parameters into Equation (1) yields Equation (4):

(4) $$\Delta k_{inc}=0.5\sigma_{inc}\sqrt{\pi d_{inc}}$$

where *d_inc_* is the inclusion size.

Assuming that the loading stress *σ* of the fatigue specimen acts uniformly on the minimum cross-sectional diameter *d*_0_ of the specimen, it is considered that, for a spherical inclusion with a diameter *d_inc_* at a depth *h_inc_* from the surface of the specimen, the stress at the boundary of the inclusion closest to the specimen surface is the stress *σ_inc_* experienced by the inclusion. Subsequently, employing linear proportionality principles, we derive Equation (5).

(5) $$\sigma_{inc}=\sigma [1-(\frac{h_{inc}-\frac{d_{inc}}{2}}{\frac{d_{0}}{2}})]$$

Substituting Equation (5) into Equation (4) results in Equation (6):

(6) $$\Delta k_{inc}=0.5\sigma [1-(\frac{h_{inc}-\frac{d_{inc}}{2}}{\frac{d_{0}}{2}})]\sqrt{\pi d_{inc}}$$

**Figure 10:**
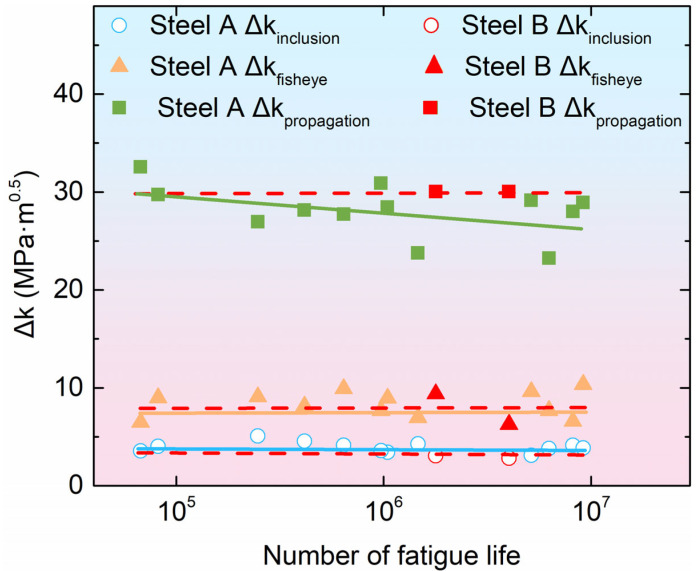
Relationship between fatigue cycles and fracture roughness.

Utilizing the fracture parameters of experimental steels A and B, which were measured in practice, the fracture parameters were substituted into Formulas (2), (3), and (6), respectively, to calculate and plot the relationship between the stress intensity factor Δ*K* and fatigue life *N_f_*, as illustrated in Figure 10. The $\Delta k_{inc}$ of the crack source (inclusion) in the two specimen sets are both 4 MPa·m^0.5^, and the $\Delta k_{fisheye}$ are both 8 MPa·m^0.5^, which are close to the fatigue crack growth threshold value Δ*K_th_* reported in references [50,51]. The $\Delta k_{propagation}$ of steel A is approximately 28 MPa·m^0.5^, and that of steel B is approximately 30 MPa·m^0.5^, which is consistent with the reported critical stress intensity factor *K_IC_* ranging from 27 to 40 in the literature [52,53]. From the standpoint of fracture process analysis, $\Delta k_{inc}$ of the inclusion is smaller than Δ*K_th_*, and a fisheye zone gradually forms around the inclusion in the source region. When the $\Delta k_{fisheye}$ corresponding to the size of the fisheye zone reaches Δ*K_th_*, the crack begins to propagate steadily. When the stress intensity factor value at the crack tip reaches the critical stress intensity factor (*K_IC_*), the crack rapidly propagates to fracture. This phenomenon elucidates the observed discrepancy in the fatigue strength of steel B and that of steel A. The two sets of specimens’ $\Delta k_{fisheye}$ of value are between $\Delta k_{inc}$ and $\Delta k_{propagation}$. It is evident that the parameters of the fisheye are dictated by the interplay of the variables *d_inc_* and *d*_3_. This finding is consistent with the results depicted in Figure 8.

Based on the aforementioned results, the *K_IC_* of Steel A is approximately 28 MPa·m^0.5^, whereas that of Steel B is approximately 30 MPa·m^0.5^. The marginally higher *K_IC_* observed for Steel B indicates a slightly superior fracture toughness compared to Steel A. This finding is in good agreement with the tensile and impact test results presented in Section 3.4.

The crack growth formula satisfies Paris’ law, and the fisheye region also conforms to this law [54], that is, Equation (7):

(7) $$\frac{da}{dN}=C{\left(\Delta K\right)}^{m}$$

where *N* is fatigue life, a is crack length, C is a constant, and m is the Paris exponent (typically 3 [55]). Integrating Equation (7) yields a result that incorporates the characteristic size of the crack, *d*, the fatigue life, *N*, and the fatigue stress, *σ*, as demonstrated in Equation (8):

(8) $$N_{f}=\frac{1-{\left(\frac{d_{inc}}{d}\right)}^{\frac{m}{2}-1}}{C{\left(0.5\sigma \sqrt{\pi }\right)}^{m}(\frac{m}{2}-1){d_{inc}}^{\frac{m}{2}-1}}$$

Assuming m = 3 and *d* ≫ *d_inc_*, the simplified form becomes Equation (9):

(9) $$N_{f}=\frac{1}{0.348C\sigma^{3}{d_{inc}}^{0.5}}$$

**Figure 11:**
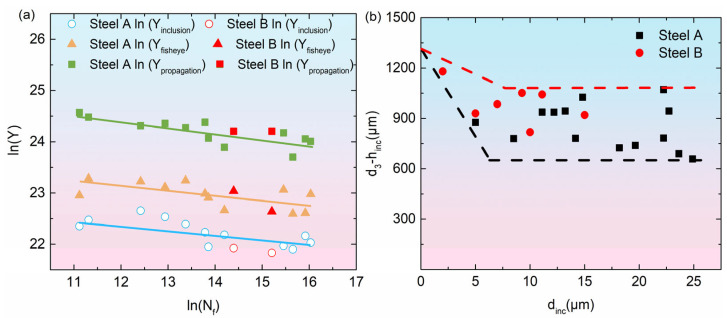
Relationship between crack propagation rate and distance: (**a**) ln(Y) and ln(*N_f_*); (**b**) crack extension and *d_inc_*; (**a**) Fit line: In(*Nf*) = (−0.1202) × ln(Y_propagation_) + 25.85, Pearson’s R: −0.8076; In(*Nf*) = (−0.0857) × ln(Y_fisheye_) + 24.15, Pearson’s R: −0.6074; In(*Nf*) = (−0.11) × ln(Y_inclusion_) + 23.76, Pearson’s R: −0.7576.

Setting $Y=F\sigma^{3}{\left(\pi d\right)}^{0.5}$ and calculating with the measured fracture parameters, the relationship between ln(Y) and ln(*N_f_*) for inclusions, fisheyes, and crack extension in steels A and B is shown in Figure 11a. For both steels, the parameter ln(Y) was found to decrease as ln(*N_f_*) increased, indicating a reduction in the rate of crack growth and an increase in the fatigue life. The distance of crack propagation from the inclusion center is given by (*d*_3_-*h_inc_*). The data presented in Figure 11b demonstrates a clear relationship between crack extension and *d*_inc_. It is evident that as *d_inc_* increases, crack extension decreases, reaching a plateau at approximately *d*_inc_ = 7.5 μm. This experimental result is consistent with Equation (9) and further demonstrates that fatigue life *N_f_* can be predicted through the inclusion size *d_inc_*.

The results obtained demonstrate quantitative relationships among applied stress, inclusion size, crack-tip stress intensity factor (Equation (6)), and fatigue life (Equation (9)).

## 5. Conclusions

In light of the aforementioned analysis, the primary conclusions that can be drawn are as follows:

1. H13 steels produced by electroslag remelting (ESR) and vacuum induction melting + vacuum arc remelting (VIM+VAR) exhibit similar grain size and microstructure. A comparison of the two samples reveals that they contain the same types of inclusions (composite inclusions of oxides and sulfides) and carbides (VC and M23C6). However, a significant disparity is observed in their distribution and size: ESR steel contains approximately four times more inclusions (>2 μm) and more than 20 times more carbides than VIM+VAR steel. The mean Feret diameter of inclusions/carbides was found to be approximately 4.5 μm in ESR steel and approximately 3 μm in VIM+VAR steel.
2. The fatigue fracture surfaces of both steels exhibit four distinct zones: crack origin (inclusion), fisheye, propagation, and final fracture. Oxide sulfide composite inclusions have been observed to function as crack initiators, with fisheye size increasing in proportion to inclusion depth.
3. The findings of the rotating bending fatigue tests and fracture parameter analysis suggest that the fatigue life of the material is predominantly influenced by the applied stress and the dimensions of the inclusion. It was observed that as the stress and the inclusion size increased, the life of the material decreased. It is found that ESR steel has numerous and large inclusions, and the crack propagation length is 18 μm~885 μm, while VIM+VAR steel has a few small inclusions, and the crack propagation length is 10 μm~1063 μm. The rotational bending strength and fatigue life of VIM+VAR steel are higher than ESR steel.
4. Equations derived from the Paris law have been demonstrated to relate inclusion size to crack-tip stress intensity factor (*K_IC_*). The *K_IC_* of ESR steel is approximately 28 MPa·m^0.5^, whereas that of VIM+VAR steel is approximately 30 MPa·m^0.5^. It can explain the H13 steel fatigue fracture process. Moreover, the relationship between inclusion size, loading stress, and fatigue life is deduced. The formula is $N_{f}=\frac{1}{0.348C\sigma^{3}{d_{inc}}^{0.5}}$. It provides a basis for predicting the rotation bending fatigue life of H13 steel.

## Figures and Tables

**Table 1:** Chemical composition of H13 hot working die steel.

	C	Si	Mn	P	S	Cr	Mo	V	Ti	T.O	N
Steel A	0.42	1.14	0.41	0.016	0.0009	4.96	1.36	0.98	0.0095	0.0016	0.015
Steel B	0.38	1.09	0.38	0.005	0.0006	5.10	1.31	0.93	0.0100	0.0008	0.001

**Table 2:** Result of phase analysis.

Steel	Precipitate	Crystal System	Lattice Constant (nm)	Element Content in Different Phases of Steel (Mass Fraction, %)				
				Cr	Fe	Mo	V	Σ
A	MC	FCC	a_0_ = 0.417	0.193	0.049	0.135	0.799	1.176
	M23C6	FCC	a_0_ = 1.070	0.732	0.178	0.054	0.046	1.010
B	MC	FCC	a_0_ = 0.417	0.187	0.043	0.137	0.774	1.141
	M23C6	FCC	a_0_ = 1.070	0.729	0.196	0.047	0.043	1.015

**Table 3:** Quantitative analysis of H13 ≥ 2 μm inclusion and carbide of steel A and B.

Specimen		Average Feret Size (μm)	Aspect (Feret Max/Feret Min)	Density (Features/mm^2^)	Index * (%)
A	Inclusion	4.5	1.4	8	0.01183
	Carbide	4.6	1.7	26	0.02282
B	Inclusion	3.0	1.4	2	0.00080
	Carbide	2.9	1.7	0	0.00003

* Index = particle area/scanned area × 100% and the scanned area = 160 mm^2^.

## Data Availability

The original contributions presented in this study are included in the article. Further inquiries can be directed to the corresponding author.
